# Achilles tenodesis for calcaneal insufficiency avulsion fractures associated with diabetes mellitus

**DOI:** 10.1186/s13018-017-0695-8

**Published:** 2017-12-13

**Authors:** Youngrak Choi, Young-woo Kwon, Young-suk Sim, Taeho Kim, Dayoung Song, Soohyun Lee

**Affiliations:** 1 Department of Orthopedic Surgery, CHA Bundang Medical Center, CHA University, 16, Yatap-ro 65-beon-gil, Bundang-gu,, Sungnam-si, Gyunggi-do 13497 Republic of Korea; 2 School of Medicine, CHA University, 120, Haeryong-ro, Pocheon-si, Gyeonggi-do Republic of Korea

**Keywords:** Calcaneal avulsion fracture, Insufficiency fracture, Achilles tenodesis, Charcot Neuroarthropathy

## Abstract

**Background:**

Calcaneal insufficiency avulsion (CIA) fractures often present with neuropathic etiology, such as Charcot neuroarthropathy (CN). Under the same surgical procedures, the outcomes of CIA fractures are less desirable, compared to the outcomes of the traumatic calcaneal avulsion fractures. Here, the study suggests Achilles tenodesis technique using suture anchor after resection of the CIA fracture fragments could provide satisfactory clinical results in the cases of surgically indicated CIA fractures.

**Materials and methods:**

This retrospective study included seven patients of calcaneal avulsion fracture who had underlying diabetes mellitus (DM) and no specific traumatic event. The patients were treated with Achilles tenodesis techniques for their CIA fractures. Achilles tenodesis was performed using suture anchor with removal of the fracture fragments. The patients were evaluated with the Foot and Ankle Outcome Score (FAOS), visual analogue scale (VAS), single-heel rise test, and X-ray images on their final follow-ups.

**Results:**

Initially, three of the CIA fracture cases treated with traditional open reduction and internal fixation reported pullout failure. Consequently, all patients received Achilles tenodesis using suture anchor after bone fragment resection and had good clinical outcomes. Only one subject with low compliance reported poor outcome.

The FAOS of each patient were obtained at a mean of 16.3 months after surgery. The results are as follows: pain 80.6 (SD = 6.2), symptom 83.8 (SD = 4.9), activities of daily living 80.5 (SD = 8.0), sport and recreation function 75.6 (SD = 11.93), and foot- and ankle-related quality of life 77.9 (SD = 6.7). On their final follow-ups, the average VAS was 2.6 (range, 1 to 4).

**Conclusion:**

Achilles tenodesis using suture anchor after bone fragment resection achieved competent clinical results in the patients with CIA fractures. The study proposes that this surgical procedure could be an appropriate treatment option for patients with CIA fractures.

**Trial registration:**

The study was approved by the institutional review board (IRB) of our medical center (IRB File No. 2016-07-043), retrospectively registered.

## Background

Calcaneal avulsion fracture is an uncommon fracture, and calcaneal insufficiency avulsion (CIA) fracture associated with Charcot neuroarthropathy (CN) is particularly unusual [1, 2]. CIA fracture is caused by normal or physiologic activity and is common in elderly diabetic patients [3]. If there is no significant trauma history or an irregular fragment, the possible etiology of the fracture becomes neuropathy or intrinsic tightness of the gastrocnemius muscle [4, 5]. The concept of CIA fractures and their association with CN was first introduced in 1991 by Kathol et al. [6]. Since then, CIA fractures are further classified into Sanders/Frykberg type V or Brodsky type 3b CN [7]. A study of Biehl W et al. reported avulsion of a calcaneal tuberosity could be an initial presentation of an underlying neuropathy [4]. Spontaneous calcaneal fractures in diabetes mellitus (DM) patients have been reported by several studies in the literature [6, 8, 9]. Decreased pain sensation and proprioception leaves the legs without protection from repeated microtrauma of the pull of the calcaneal tendon [4].

Until now, CIA fractures are commonly treated with open reduction and internal fixation in the same manner as treating the traumatic calcaneal avulsion fractures [4, 6, 10, 11]. The conventional surgical approaches for calcaneal avulsion fractures are fixation with cancellous screws, K wires, and tension band wiring [12]. There has yet been a surgical procedure established specifically for the CIA fractures, since relatively poor surgical outcomes are seen in these cases, under the same procedure [13].

The purpose of this study is to report the clinical outcome of seven patients of CIA fracture treated with Achilles tenodesis using suture anchor after resection of the fragment. We hypothesized that the patients with CN had a lower bone healing potential, so that fracture fragment resection and fixation with multiple anchors would have a stronger fixation power.

## Methods

### General information

**Fig. 1:**
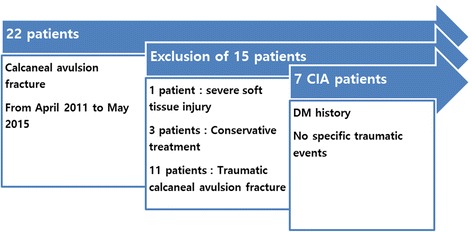
Patient selection process including inclusion and exclusion

This retrospective study initially included 22 patients who were diagnosed with calcaneal avulsion fracture in a single institution from April 2011 to May 2015. The study was approved by the institutional review board (IRB) of our medical center. One patient was excluded because he had severe concomitant traumatic soft tissue injury. Three patients who underwent conservative treatment for their calcaneal avulsion fractures due to poor general health condition were excluded. Eleven patients did not have DM and had a trauma history and were excluded as traumatic calcaneal avulsion fractures. Finally, seven patients (four males and three females) with an average age of 55.3 years (range, 43 to 79 years) were studied. All patients had diabetes (average disease period, 16.7 years) and reported no specific traumatic events (Fig. 1). The mechanisms of their injuries were low-energy traumas, such as walking or climbing a hill. Eleven patients without diabetes had histories of traumatic events (Table 1).

**Table 1:** Demographic data of patients

No.	Sex	Age	Period of follow-up (months)	PMHx	Duration of DM (years)	HgbA1c	Injury mechanism
1	F	63	14	DM	20	7.6	Ankle sprain
2	M	55	15	DM ESRD	10	8.5	Walking
3	F	48	20	DM	6	15.1	Walking
4	M	51	18	DM	16	7.4	Climbing a hill
5	F	48	18	DM ESRD	25	8.9	Climbing a hill

Preoperative radiographs of the lateral and axial view of the calcaneus, anteroposterior and oblique views of the foot, and CT scans with three-dimensional reconstruction were taken for each subject. The visual analogue scale (VAS) was used to rate the level of pain caused by fracture. The preoperative average VAS was 3.7 (range, 2 to 6).

The postoperative follow-up period ranged from 13 to 20 months (average, 16.3 months). On their final follow-ups, patients were evaluated with the Foot and Ankle Outcome Score (FAOS), VAS, single-heel rise test, and X-ray images.

### Surgical technique

**Fig. 2:**
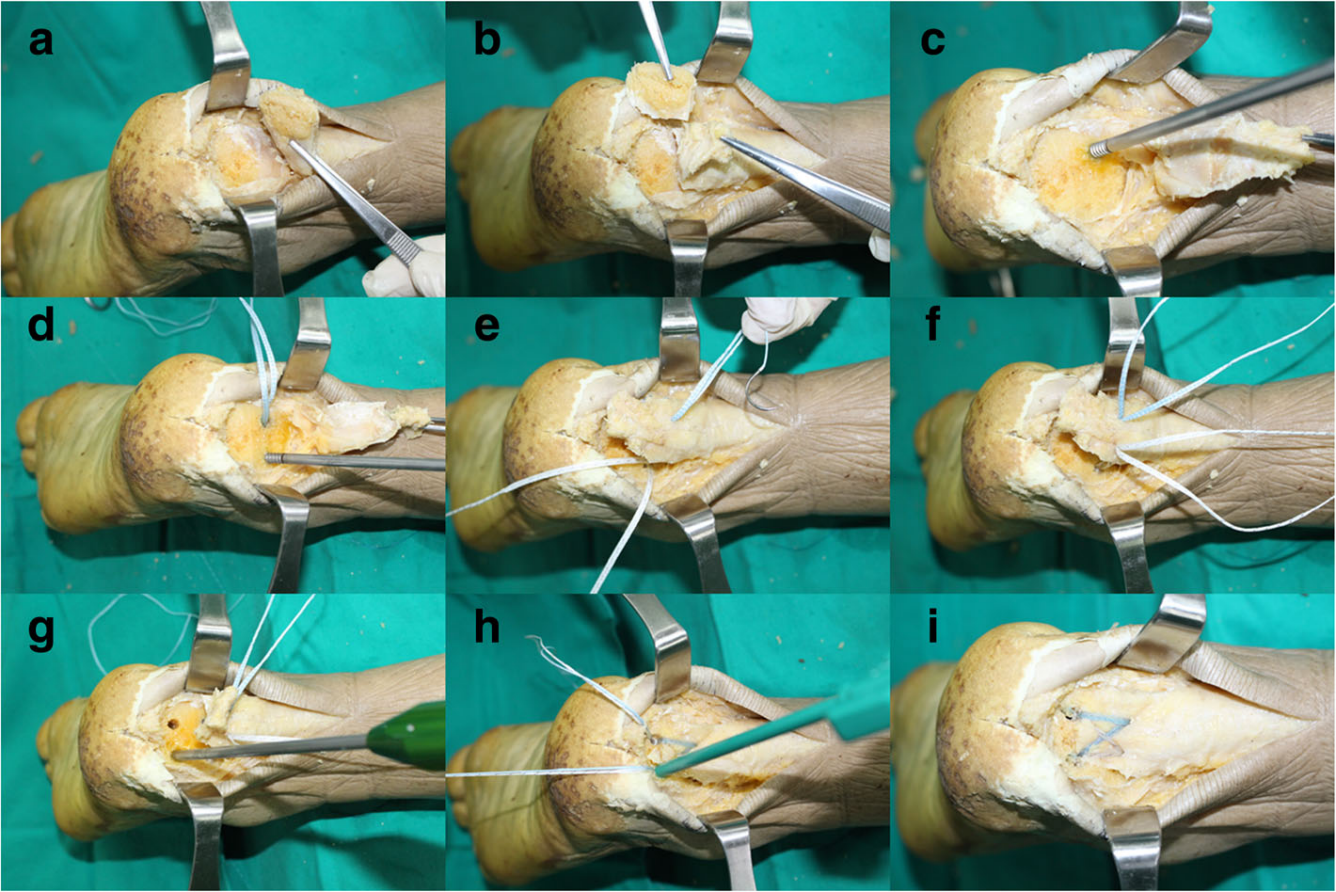
Achilles tenodesis using suture anchor after bone fragment resection. The Achilles tendon insertion site was exposed (**a**) and all fracture fragments were excised (**b**). The drill for the medial anchor was drilled into the fifth metatarsal (**c**) and the drill for the lateral anchor was tilted towards the first metatarsal (**d**). The tail of a FiberLink™ suture was passed, for use as a shuttle, through the Achilles tendon (**e**) and shuttled the FiberTapes (**f**). A bone socket is prepared using a punch (**g**). Tension is applied to the FiberTapes so that the tissue is reduced and compressed against the bone (**h**). The driver is completely advanced into the bone socket, until the anchor body contacts the bone (**i**)

**Fig. 3:**
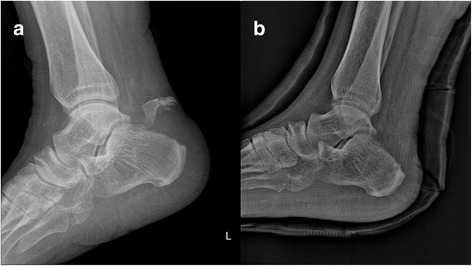
A calcaneal avulsion fracture in the preoperative X-ray (**a**) and the fragment attached to the Achilles tendon removed in the postoperative X-ray (**b**)

Single orthopedic surgeon performed all surgeries. In the operating room, the patient was placed in prone position with a support under their lower legs to allow free foot movement. A pneumatic tourniquet was applied to the thigh. A longitudinal incision of 7–8 cm with minimal undermining was made. The insertion site of the Achilles tendon (Fig. 2a) was first exposed, then all fracture fragments were excised (Fig. 2b). The Achilles tendon was reattached to the remaining calcaneal tuberosity using the SpeedBridge™ (Arthrex, Naples, FL) double-row anchor system. To place the medial anchor, a hole was drilled into the fifth metatarsal bone (Fig. 2c). To place the lateral anchor, another hole was drilled into the first metatarsal with an angle (Fig. 2d). The BioComposite™ SwiveLock C was inserted into each of the prepared bone sockets until the anchor body makes a contact with the bone. The tail of a FiberLink™ suture was passed through the Achilles tendon to be used as a shuttle (Fig. 2e). FiberLink tail was then pulled out to shuttle the FiberTapes through the hole made in the Achilles tendon (Fig. 2f). One FiberTape tail was retrieved from each of the anchors, and it was preloaded through the SwiveLock C eyelet. The bone sockets were prepared using a punch. The anchor was generally positioned at 1.5 cm distal from the resection site (Fig. 2g). The eyelet of the implant was brought to the edge of the bone socket, and the slack was individually removed from each of the FiberTape limbs. Tension was applied to the FiberTapes to make sure that the tissue is reduced and compressed against the bone (Fig. 2h). The driver was applied completely into the bone socket to advance beyond the first laser line until the anchor body makes contact with the bone (Fig. 2i). After saline lavage, paratenon and subcutaneous layers were closed with absorbable no. 3/0 sutures. Non-absorbable sutures were used for skin closure. The previously observed facture fragments of the calcaneal avulsion fracture were no longer seen in the postoperative X-ray images (Fig. 3).

### Postoperative management

A short leg splint was applied for 3 weeks with 30° of ankle plantar flexion. All weight-bearing exercises were restricted. In the third postoperative week, tolerable weight bearing with a walking brace and functional exercises of the ankle were initiated. At this time, ankle dorsiflexion was gradually increased by 10°. The patient began full-load ambulation with neutral ankle position at 6 weeks after surgery.

### Functional assessment

The FAOS was used to assess and compare the ankle function before injury and at final follow-up. The FAOS is a 42-item questionnaire that assesses the relevant outcome of the patient in five separate subscales (pain, other symptoms, activities of daily living, sport and recreation function, foot and ankle-related quality of life). The total score was 100 for this scale.

## Results

Initially, three of the seven patients underwent open reduction and internal fixation (ORIF) using cannulated cancellous screws or tension band wiring (TBW), while the remaining four patients received Achilles tenodesis using suture anchor after bone fragment resection. However, complications such as pullout failures and wound problems occurred in three patients who underwent ORIF. These patients received revisional Achilles tenodesis. Consequently, all patients received Achilles tenodesis using suture anchor after bone fragment resection (Table 2).

**Table 2:** The treatment process of patients

	No.	1st operation	2nd operation	3rd operation
CIA fractures	1	ORIF^a^ with screw	ORIF with TBW^b^	Achilles tenodesis and bone fragment resection
	2	Achilles tenodesis and bone fragment resection	Suture anchor removal because of pullout failure	
	3	ORIF with screw	Achilles tenodesis and bone fragment resection	
	4	ORIF with screw	Achilles tenodesis and bone fragment resection	
	5	Achilles tenodesis and bone fragment resection		

^a^Open reduction and internal fixation

^b^Tension band wiring

FAOS for each patient were obtained at a mean of 16.3 months after surgery, and the scores are as follows: pain 80.6 (SD = 6.2), symptom 83.8 (SD = 4.9), activities of daily living 80.5 (SD = 8.0), sport and recreation function 75.6 (SD = 11.93), and foot- and ankle-related quality of life 77.9 (SD = 6.7). On their final follow-ups, the average VAS was 2.6 (range, 1 to 4). Only one patient reported negative for the single heel rise test (Table 3).

**Table 3:** Foot and Ankle Outcome Score (FAOS), visual analogue scale (VAS), and single-heel rise test of patients at final follow-up

		FAOS						
Group	No.	Pain	Symptoms	ADL^a^	SPORT&REC^b^	QOL^c^	VAS	Single-heel rise test
CIA fractures	1	77.7	82.1	83.8	75	81.2	3	+
	2	69.4	57.1	59.1	45	58.7	4	–
	3	88.8	89.2	83.8	85	87.5	3	+
	4	71.1	80.7	76.1	75	70	2	+
	5	79.4	80.5	61.7	70	78.7	3	+
	6	80.5	89.2	82.3	75	75	2	+
	Mean	77.8	79.8	74.5	70.9	75.2	2.8	

^a^Activities of daily living

^b^Sport and recreation function

^c^Foot and ankle-related quality of life

The clinical outcomes of Achilles tenodesis in the cases of CIA fractures were satisfactory, except for 1 subject (case no. 2) who reported pullout of the suture anchors. The patient disregarded the advice to avoid weight bearing during the first three postoperative weeks and walked with full weight. No other complications were reported in this study.

## Discussion

The CIA fractures caused by CN are rare; yet, the management may be demanding due to the low healing potential the patients accompany. These fractures have been observed with increasing frequency in patients with diabetes due to the close correlation between the insufficiency fractures and peripheral neuropathy [4, 6]. Several studies indicated the decrease in bone quality in patients with CN [14]. Poor bone quality raises the possibility to have failed fixation with cancellous screw and bone healing. Besides, Ramaswamy et al. concluded in their study that the holding power of the screws is directly proportional to density of the bone [15]. Poor bone stock, lack of proper bone for screw, and the presence of related pathology make these repairs susceptible to surgical failure [16].

Traditionally, CIA fractures have been treated with open reduction and internal fixation, in the same manner as the nondiabetic calcaneal avulsion fractures. Diverse fixation methods such as cancellous screws, K wires, tension band wiring (TBW), and locking plates are available to surgically manage the calcaneal avulsion fractures. However, CIA fractures result in poor outcomes when operated in the same way as the traumatic cases of calcaneal avulsion fracture, since higher incidence of infection, nonunion, malunion, failure in fixation, and delayed wound healing are observed in these cases [13]. Initially, cancellous screws or TBW were tried for the cases of CIA fracture at our institution, but both screws and wires pulled out and failed eventually. This may be attributed to the reduced regenerative potential and poor bone quality of the CN patients, as mentioned above.

Recently, a fixation technique using suture anchor have been introduced; however, surgical outcomes of the technique have yet been established [2, 6, 11]. Yoshida et al. treated a case of calcaneal avulsion fracture managed with a soft anchor and lag screw technique [17]. Greenhagen et al. reported a case which successfully treated the CIA injury using the Achilles suture bridge technique, as well [18]. In both cases, the suture anchor fixation was shown to be superior to the traditional screw fixation, probably due to the decrease in bone mineral density (BMD) [17, 18]. Thus, this study further obtained the results of seven CIA fracture cases and compared them to the cases of traumatic calcaneal avulsion fractures. The study not only introduces a surgical technique but also demonstrates the competence of a new technique by investigating in multiple cases.

The technique of tenodesis with suture anchor after fracture fragment removal was able to achieve direct fixation of the tendon to the remaining bone. This could avoid the failure of bone-to-bone fixation and increase the fixation strength of the Achilles tendon by establishing maximal contact between the tendon and the bone. Lin et al. suggested in their cadaveric study of fracture of the greater tuberosity of the humerus that the soft anchor bridge technique provides greater biomechanical strength than that of screws [19]. To solve the issue of limited space available for fixation, several anchors that are smaller than the screws can be applied to increase the clamping force.

**Fig. 4:**
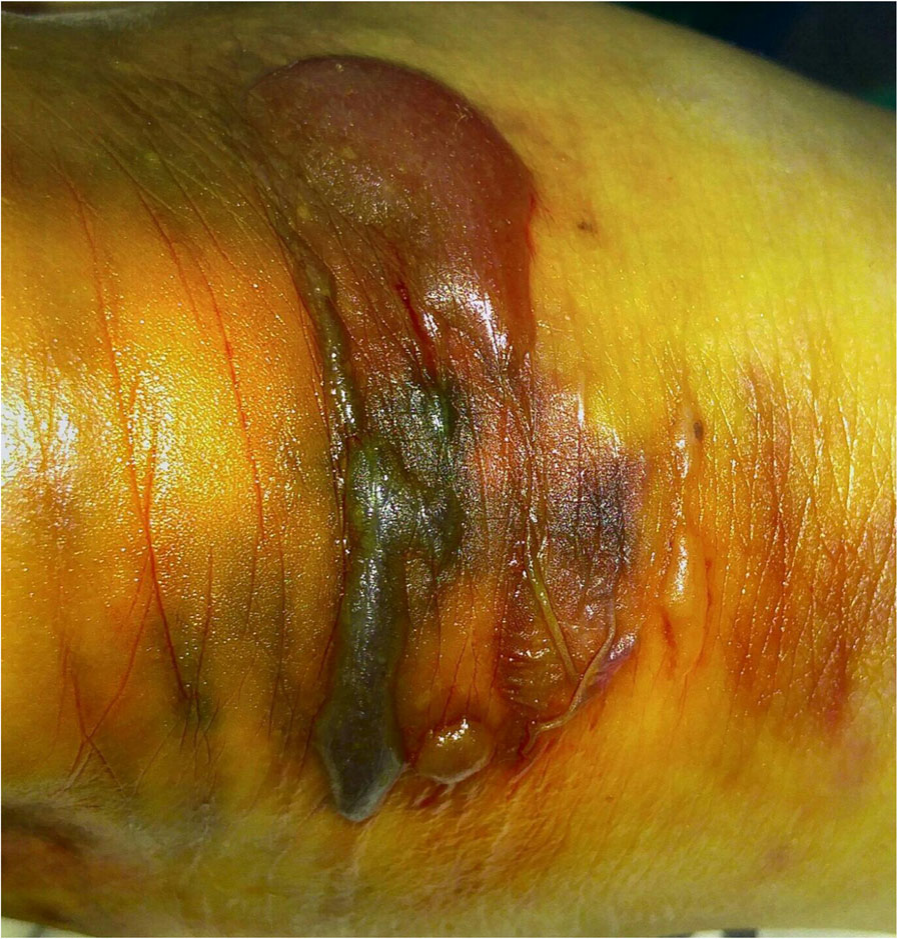
Hemorrhagic bullae after 1 day of trauma

In case of calcaneal avulsion fracture, the posterior skin of the heel must be evaluated quickly to check for skin tenting. If skin tenting is observed, the fracture must be immediately reduced and fixed since such cases are at risk of skin necrosis [20]. Skin necrosis and wound problem are frequently reported, especially in diabetic calcaneal avulsion fractures. In this study, two patients reported initial skin problems such as blisters due to compression by the bone fragments (Fig. 4), yet the problems resolved after surgery. In these cases, resection of the fracture fragment reduced wound complications by eliminating the time required for fracture reduction and decreased the compression pressure applied on the surgical site.

One of the limitations of this study is that three CIA patients did not receive Achilles tenodesis for their primary operation. These patients were operated on at the beginning of the study period and may not be appropriate to be compared with patients who underwent primary tenodesis. However, it can be interpreted that similar results are obtained with other studies showing poor results when treating CIA fractures by conventional methods. Another limitation is the small number of cases due to the low incidence of CIA fractures. CIA fracture is a rare disease that is difficult to diagnose because it is not typical of pain. If more cases are investigated, the adequacy of the new technique to treat CIA fractures can be more thoroughly assessed.

## Conclusion

A calcaneal avulsion fracture of a patient with a previous history of diabetes mellitus and no history of trauma may be considered as a CIA fracture associated with CN and the result of conventional ORIF is not good. Achilles tenodesis using suture anchor after bone fragment resection in patients with CIA can achieve satisfactory clinical outcomes, suggesting this technique as an appropriate treatment option for the patients with CIA fractures.

## Abbreviations

BMD: Bone mineral density
CIA: Calcaneal insufficiency avulsion
CN: Charcot neuroarthropathy
DM: Diabetes mellitus
FAOS: Foot and Ankle Outcome Score
ORIF: Open reduction and internal fixation
TBW: Tension band wiring
VAS: Visual analogue scale
