# Correction to: Achilles tenodesis for calcaneal insufficiency avulsion fractures associated with diabetes mellitus

**DOI:** 10.1186/s13018-018-0744-y

**Published:** 2018-03-02

**Authors:** Youngrak Choi, Young-woo Kwon, Young-suk Sim, Taeho Kim, Dayoung Song, Soohyun Lee

**Affiliations:** 1 Department of Orthopedic Surgery, CHA Bundang Medical Center, CHA University, 16, Yatap-ro 65-beon-gil, Bundang-gu, Sungnam-si, Gyunggi-do 13497 Republic of Korea; 2 School of Medicine, CHA University, 120, Haeryong-ro, Pocheon-si, Gyeonggi-do Republic of Korea

## Correction

The original publication of this article [1] contained the wrong versions of Tables 1, 2 and 3. In this correction the updated tables are published. The original publication has been updated.

**Table 1:** Demographic Data of Patients

	No	Sex	Age	Period of follow-up (months)	PMHx	Duration of DM (years)	HgbA1c	Injury Mechanism	Pre-operative VAS
**CIA fracture**	**1**	F	63	14	DM	20	7.6	Ankle sprain	2
	**2**	M	55	15	DM ESRD	10	8.5	Walking	3
	**3**	F	48	20	DM	6	15.1	Walking	6
	**4**	M	51	18	DM	16	7.4	Climbing a hill	4
	**5**	F	48	18	DM ESRD	25	8.9	Climbing a hill	5
	**6**	M	43	16	DM	15	8.1	Walking	3
	**7**	M	79	20	DM ESRD	25	9.1	Walking	3

**Table 2:** The treatment process of Patients

	No	1^st^ operation	2^nd^ operation	3^rd^ operation
**CIA fracture**	1	ORIF^a^ with screw (pulled out)	ORIF with TBW^b^ (pulled out)	**Achilles tenodesis** **and bone fragment resection**
	2	**Achilles tenodesis** **and bone fragment resection**	Suture anchor removal because of pulled out failure	
	3	ORIF with screw (pulled out)	**Achilles tenodesis** **and bone fragment resection**	
	4	ORIF with screw (pulled out)	**Achilles tenodesis** **and bone fragment resection**	
	5	**Achilles tenodesis** **and bone fragment resection**		
	6	**Achilles tenodesis** **and bone fragment resection**		
	7	**Achilles tenodesis** **and bone fragment resection**		

^a^Open reduction and internal fixation

^b^Tension band wiring

**Table 3:** Foot and Ankle Outcome Score (FAOS), Visual Analogue Scale (VAS) and single-heel rise test of Patients at final follow up

		FAOS						
Group	No	PAIN	SYMPTOMS	ADL^a^	SPORT&REC^b^	QOL^c^	VAS	Single-heel rise test
**CIA fractures**	1	77.8	82.1	85.3	75	81.3	3	+
	2	72.2	75.0	63.2	45	68.8	4	-
	3	88.9	89.3	85.3	75	87.5	3	+
	4	72.2	82.1	80.9	85	75	2	+
	5	77.8	82.1	77.9	80	81.3	3	+
	6	85.6	89.4	86.9	85	75	2	+
	7	89.5	86.9	83.8	85	76.4	1	+
	**Mean**	**80.6**	**83.8**	**80.5**	**75.6**	**77.9**	**2.6**	

^a^Activities of daily living

^b^Sport and recreation function

^c^Foot and ankle-related quality of life
